# Differential Response of Chondrocytes and Chondrogenic-Induced Mesenchymal Stem Cells to C1-OH Tributanoylated *N*-Acetylhexosamines

**DOI:** 10.1371/journal.pone.0058899

**Published:** 2013-03-14

**Authors:** Jeannine M. Coburn, Nicholas Bernstein, Rahul Bhattacharya, Udayanath Aich, Kevin J. Yarema, Jennifer H. Elisseeff

**Affiliations:** 1 Translational Tissue Engineering Center, Wilmer Eye Institute and the Department of Biomedical Engineering, Johns Hopkins University, Baltimore, Maryland, United States of America; 2 Department of Chemical and Biomolecular Engineering, Johns Hopkins University, Baltimore, Maryland, United States of America; University of Minho, Portugal

## Abstract

Articular cartilage has a limited ability to self-repair because of its avascular nature and the low mitotic activity of the residing chondrocytes. There remains a significant need to develop therapeutic strategies to increase the regenerative capacity of cells that could repair cartilage. Multiple cell types, including chondrocytes and mesenchymal stem cells, have roles in articular cartilage regeneration. In this study, we evaluated a platform technology of multiple functionalized hexosamines, namely 3,4,6-*O*-tributanoylated-*N*-acetylgalactosamine (3,4,6-*O*-Bu_3_GalNAc), 3,4,6-*O*-tributanoylated-*N*-acetylmannosamine (3,4,6-*O*-Bu_3_ManNAc) and 3,4,6-*O*-Bu_3_GlcNAc, with the potential ability to reduce NFκB activity. Exposure of IL-1β-stimulated chondrocytes to the hexosamine analogs resulted in increased expression of ECM molecules and a corresponding improvement in cartilage-specific ECM accumulation. The greatest ECM accumulation was observed with 3,4,6-*O*-Bu_3_GalNAc. In contrast, mesenchymal stem cells (MSCs) exposed to 3,4,6-*O*-Bu_3_GalNAc exhibited a dose dependent decrease in chondrogenic differentation as indicated by decreased ECM accumulation. These studies established the disease modification potential of a hexosamine analog platform on IL-1β-stimulated chondrocytes. We determined that the modified hexosamine with the greatest potential for disease modification is 3,4,6-*O*-Bu_3_GalNAc. This effect was distinctly different with 3,4,6-*O*-Bu_3_GalNAc exposure to chondrogenic-induced MSCs, where a decrease in ECM accumulation and differentiation was observed. Furthermore, these studies suggest that NFκB pathway plays a complex role cartilage repair.

## Introduction

Articular cartilage covers the surfaces of all diarthroidal joints. It is a highly hydrated tissue that serves to distribute loads to bone ends and facilitates near frictionless movement [1]. Damage to articular cartilage via trauma or disease is a clinical challenge, as the tissue has limited ability to self-repair, leaving it vulnerable to further degeneration. There are two cell types commonly investigated in the repair of cartilage: chondrocytes and mesenchymal stem cells (MSCs). Chondrocytes are the resident cells in articular cartilage. They produce the extracellular matrix (ECM) and release degradation enzymes to remove aging matrix, maintaining tissue homeostasis. MSCs are multipotent stem cells with the ability to differentiate into multiple cell lineages, including chondrocytes, and reside within the bone marrow [2]. A common surgical strategy used to repair articular cartilage defects, microfracture, utilizes MSCs naturally residing within the bone marrow as a cell source for tissue repair [3].

Damage to diarthroidal joint tissue, whether due to sports injuries or overuse, causes early-onset osteoarthritis (OA) through multiple mechanisms, including joint instability that causes irregular force distribution [4], [5] and local inflammation [6], [7]. To date, there are no disease-modifying drugs for effective management or treatment of OA. Preventative theraupetics that reduce the progressive cartilage damage after injury could potentially decrease the prevalence of early-onset OA. Local inflammation is a potential therapeutic target, as inflammation results in increased levels of ECM degradation enzymes. Inflammation can be driven by inflammatory cytokines or other stimuli. The inflammatory process triggers increased expression of pro-inflammatory cytokines creating a positive feedback loop and further perturbs joint space homeostasis. Nuclear factor-κB (NFκB) is a transcription factor that regulates the expression of inflammatory cytokines and degradation enzymes implicated in OA [7]. As NFκB is a central transcription factor in the inflammatory process, inhibition of NFκB activity has been proposed as a potential therapeutic target [8].

**Figure 1:**
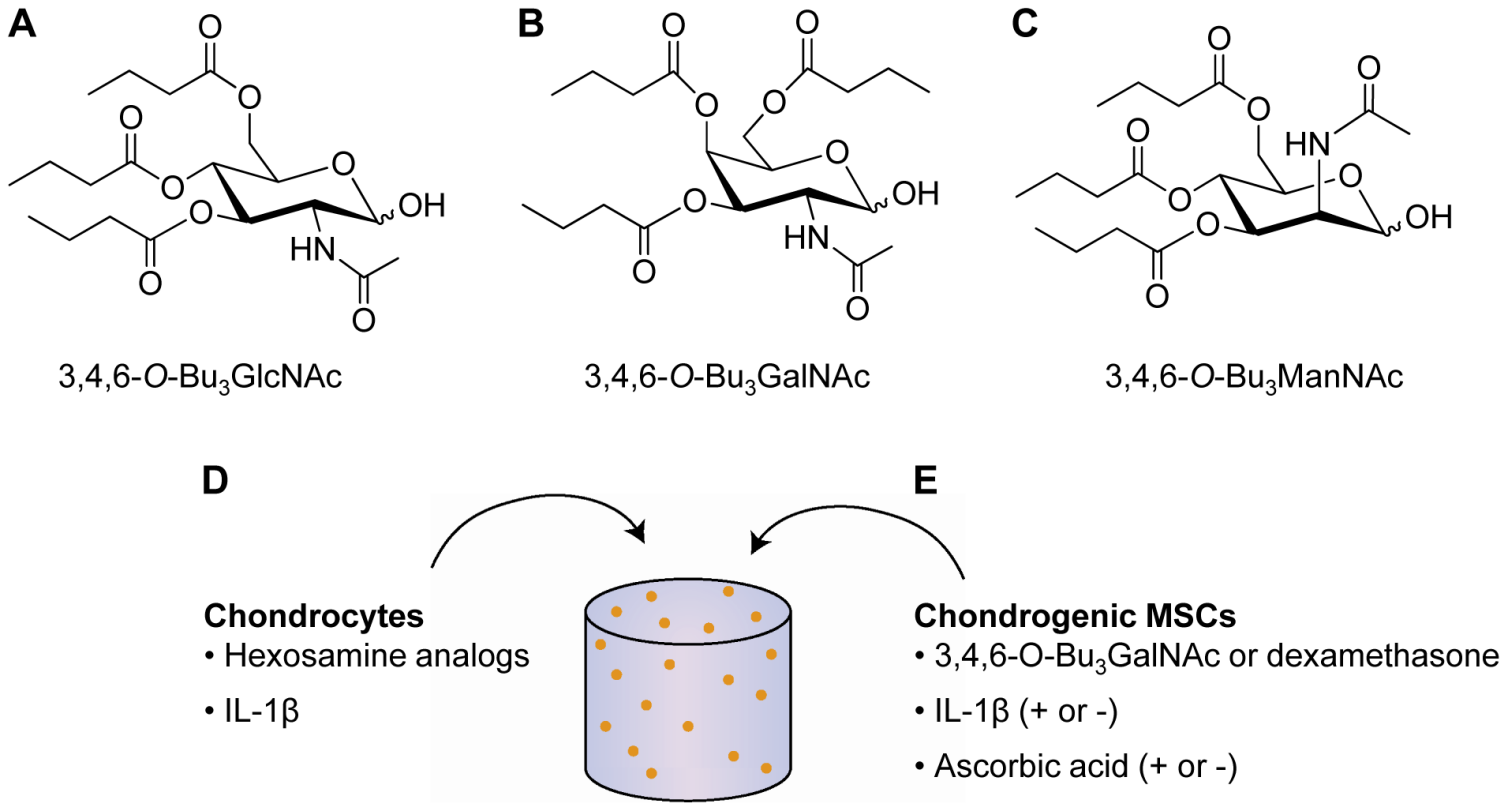
Outline of experimental design. Chemical structures of C1-OH tributanoylated hexosamines, (A) *N*-acetylglucosamine (GlcNAc-a), (B) *N*-acetylgalactosamine (GalNAc-a) and (C) *N*-acetylmanosamine (ManNAc-a). (D) Chondrocytes and (E) chondrogenic-induced MSCs were cultured in 3D poly(ethylene glycol)-diacrylate (PEGDA) hydrogels. (D) IL-1β-stimulated chondrocytes were exposed to varying concentrations of each of the three analogs in separate experiments. (E) Chondrogenic-induced MSCs were cultured with or without IL-1β in combination with varying concentrations of GalNAc-a or 100 nM dexamethasone.

In the present work, we investigated a platform technology containing multiple *N-*acetylhexosamines functionalized in a similar manner, specifically 3,4,6-*O*-Bu_3_GalNAc, 3,4,6-*O*-Bu_3_ManNAc and 3,4,6-*O*-Bu_3_GlcNAc. The chemical structures of 3,4,6-*O*-Bu_3_GlcNAc, 3,4,6-*O*-Bu_3_GalNAc and 3,4,6-*O*-Bu_3_ManNAc are shown in Figure 1A–C; these molecules are subsequently denoted as GlcNAc-a, GalNAc-a and ManNAc-a, respectively, in this manuscript. We evaluated the general effects of these analogs on IL-1β-stimulated chondrocytes and on chondrogenic-induced MSCs to determine their potential to be developed into disease-modifying agents for treating cartilage damage. The results of this work suggest the potential of a new class of hexosamine analogs as disease modifying agents for treating cartilage damage and suggest multiple angles to explore when developing cartilage therapeutics.

## Materials and Methods

### Synthesis of Monosaccharide Hybrid Molecules

Molecules were synthesized as previously reported (3,4,6-*O*-Bu_3_ManNAc [9], 3,4,6-*O*-Bu_3_GlcNAc [10] and 3,4,6-*O*-Bu_3_GalNAc [11], denoted as ManNAc-a, GlcNAc-a and GalNAc-a, respectively). Compounds were maintained at −20°C after lyophilization. Stock solutions used for experiments were made periodically by dissolving an analog in 100% ethanol to make a final concentration of 100 mM and storing the solution at 4°C.

### Cell Isolation and Culturing

Bovine chondrocytes were isolated from 4-to 8-week-old calves (Research 87, Inc., Boylston, MA), as previously described [12]. Briefly, cartilage was dissected from the femoral patellar groove and condyles and minced into approximately 1 mm^2^ pieces. Cells were isolated from the tissue by digesting overnight at 37°C with 2 mg/ml type II collagenase (Worthington Biochemical Corp., Lakewood, NJ). The cell suspension was passed through a 70 µm cell strainer, followed by centrifugation. Cells were washed 3 times using sterile phosphate buffered saline (PBS). Chondrocyte medium was composed of high glucose Dulbecco’s Modified Eagle’s Medium (DMEM) supplemented with 10 mM HEPES, 0.4 mM L-proline, 50 µg/ml ascorbic acid, 10% (v/v) fetal bovine serum (FBS; Hyclone Laboratories, Inc., Logan, UT), 0.1 mM non-essential amino acids, 100 U/ml penicillin and 100 µg/ml streptomycin.

Goat MSCs were isolated from 2-to 4-year-old goats (Thomas D. Morris, Inc., Reisterstown, MD), as previously described [13]. Briefly, bone marrow aspirates treated with 6,000 U/ml heparin were washed twice with PBS. Cells were plated at a density of 120,000 mononuclear cells/cm^2^ and cultured in high glucose DMEM supplemented with 10% (v/v) FBS, 2 mM L-glutamine, 100 U/ml penicillin and 100 µg/ml streptomycin. The following day, tissue culture dishes were washed with PBS and medium was replaced. Medium changes were performed 3 times per week. Cells were passaged using 0.25% trypsin. They were used for experiments after passage four.

### Metabolic Activity/Toxicity Screening: Water Soluble Tetrazonium-1 (WST-1) Assay

Chondrocytes were plated in 96-well plates at 10,000 cells/well. Cells were allowed to adhere and spread for 3 days, after which medium was changed to medium containing hexosamine analogs at concentrations ranging from 0 µM-320 µM and cultured for an additional 3 days. The WST-1 assay (Roche Molecular Biochemicals, Mannheim, Germany) was used to determine cell proliferation in the presence of sugar analog following the manufacturer’s protocol. Briefly, medium was aspirated from each well and the wells were washed with sterile PBS. One hundred microliters of WST-1 working solution was added to each well and 6 empty wells for background subtraction. The WST-1 reagent was incubated with the cells for 3–4 h, during which time, the enzymatic cleavage of the tetrazolium salt WST-1 to formazan by cellular mitochondrial dehydrogenases resulted in a visible light color shift in the medium. Absorbance of the wells was read using a microplate reader at a wavelength of 450 nm. The absorbance of the wells containing WST-1 reagent with no cells was subtracted from the absorbance readings. Data is presented as the background subtracted reading normalized to 0 µM wells from five independent experiments for each hexosamine analog.

### Photoencapsulation in Poly(Ethylene Glycol)-Diacrylate (PEGDA) Hydrogels and Construct Culturing

For all 3D culture experiments, cells were photoencapsulated in PEGDA hydrogels, as previously described [13], [14]. Briefly, 100 mg PEGDA (3.4 kDa, SunBio, Anyang City, South Korea) was dissolved in 1 ml sterile PBS. A photoinitiator solution of Irgacure® 2959 (CIBA Specialty Chemicals) was prepared by dissolving 100 mg of Irgacure® 2959 in 1 ml 70% ethanol. Five microliters of the Irgacure® 2959 solution was added to 1 ml of PEGDA solution. Cells were suspended in the hydrogel precursor solution at a density of 20 million cells/ml and polymerized in sterile molds. Based on previous studies a high cell density was chosen to best support diffentiation and cell phenotype [15], [16], [17]. Polymerization was carried out using UV light (365 nm, 3.2 mW/cm^2^) exposure for 5 minutes.

For chondrocyte experiments, the cell-laden hydrogels were cultured in chondrocyte medium supplemented with 10 ng/ml IL-1β for 3 days. Following the initial 3 days, constructs were cultured with medium containing specific concentrations of hexosamine analog with 10 ng/ml IL-1β for an additional 21 days. The medium was changed 3 times per week.

For chondrogenesis experiments, the cell-laden hydrogels were cultured in chondrogenic induction medium (high glucose DMEM supplemented with 50 µg/ml ascorbic acid-2-phosphate, 40 µg/ml L-proline, 100 µg/ml sodium pyruvate, 1% ITS-premix (6.25 µg/ml insulin, 6.25 µg/ml transferrin, 6.25 ng/ml selenous acid, 1.25 mg/ml bovine serum albumin [BSA], 5.35 µg/ml linoleic acid [Collaborative Biomedical, BD Bioscience, Bedford, MA]) and 10 ng/ml TGF-β1 (Fitzgerald Industries International, Acton, MA) with specified concentrations of GalNAc-a or 100 nM dexamethasone (as a control). Constructs were cultured for 21 days with medium changes 3 times per week. For IL-1β stimulation, the cell-laden hydrogels were cultured for 3 days in chondrogenic induction medium supplemented with 10 ng/ml IL-1β. Constructs were cultured for an additional 21 days with chondrogenic induction medium supplemented with 10 ng/ml IL-1β and the specified concentration of GalNAc-a.

### Biochemical Analysis

The constructs (n = 3 for chondrocyte experiments and n = 4 for chondrogenic experiments) were lyophilized for 48 h and the dry weights measured. Constructs were then homogenized in 125 µg/ml papainase (Worthington Biochemical Corp., Lakewood, NJ) and digested for 16 h at 60°C. Quantification of DNA content was carried out using a Hoescht Dye 33342 DNA assay [18]. Calf thymus DNA was used to generate standard curves. Quantification of sulfated glycosaminoglycans (sGAG), a molecule found in cartilage ECM, was carried out using a 1,9-dimethylmethylene blue (DMMB) dye assay; chondroitin sulfate was used to generate standard curves [19]. Quantification of collagen was carried out via measuring hydroxyproline content after hydrolyzing in 6 N HCl overnight at 120 °C. The reaction of hydroxyproline with chloramine-T and p-dimethylaminobenzaldehyde was performed as a measure of total collagen content; hydroxyproline was used to generate standard curves [20].

### Histology and Immunohistochemistry

Constructs were fixed in 10% formalin, embedded in paraffin and sliced into 5 µm sections. After paraffin removal and subsequent rehydration, sections were stained for proteoglycans using Safranin-O and fast green or prepared for immunohistochemistry. For immunohistochemical staining of type II collagen, endogenous peroxidase was quenched using 3% (v/v) hydrogen peroxide in methanol. Sections were then incubated at 37°C with 2.5% (w/v) hyaluronidase and stained using a Histostain-SP Kit (AEC, Broad Spectrum) (Life Technologies, Grand Island, NY) following the manufacturer’s instructions. Primary antibody for type II collagen (Fitzgerald Industries International, Acton, MA) was used at a dilution of 1∶100 in 1% (w/v) BSA dissolved in PBS.

### RNA Isolation and Real-Time PCR

Total RNA was extracted using TRIzol^®^ reagent (Life Technologies, Grand Island, NY) following the manufacturer’s protocol. cDNA was synthesized using Superscript^®^ II reverse transcriptase (Life Technologies, Grand Island, NY) following the manufacturer’s protocol. Real-time PCR was carried out using a StepOnePlus™ Real-Time PCR System (Life Technologies, Grand Island, NY). mRNA amounts for bovine primers were calculated using the ??Ct method. Relative mRNA quantities for goat primers were calculated using the Pfaffl method [21]. The PCR primers used are listed in Tables S1 and S2.

### Statistical Analysis

Data are expressed as mean ± standard deviation (SD) for biochemical quantification and mean ± standard error of the mean (SEM) for gene expression. Statistical significance was determined by student’s *t*-test or one-way analysis of variance (ANOVA) followed by Tukey HSD test using SPSS 18.0 software (IBM, Armonk, NY). Significance was determined at P < 0.05.

## Results

### Effect of Tributanoylated Hexosamine Exposure on Monolayer Chondrocyte Viability

The chemical structures of 3,4,6-*O*-Bu_3_GlcNAc, 3,4,6-*O*-Bu_3_GalNAc, and 3,4,6-*O*-Bu_3_ManNAc are shown in Figure 1A-C; these molecules are subsequently denoted as GlcNAc-a, GalNAc-a, and ManNAc-a in this manuscript. Because of the previously reported toxicity of these compounds [11], we used the WST-1 assay to determine the dose-dependent viability of analog-treated chondrocytes so as to avoid cytotoxic levels. The analogs did not reduce cell viability at concentrations of less than 40 µM, while reduced viability was observed starting at concentrations of 80 µM for GlcNAc-a and ManNAc-a and 160 µM for GalNAc-a (Figure S1). Based on these results, we chose to perform the subsequent studies at analog concentrations up to 150 µM.

### Comparison of ECM Production by IL-1β-Stimulated Chondrocytes Exposed to Tributanoylated Hexosamine

**Figure 2:**
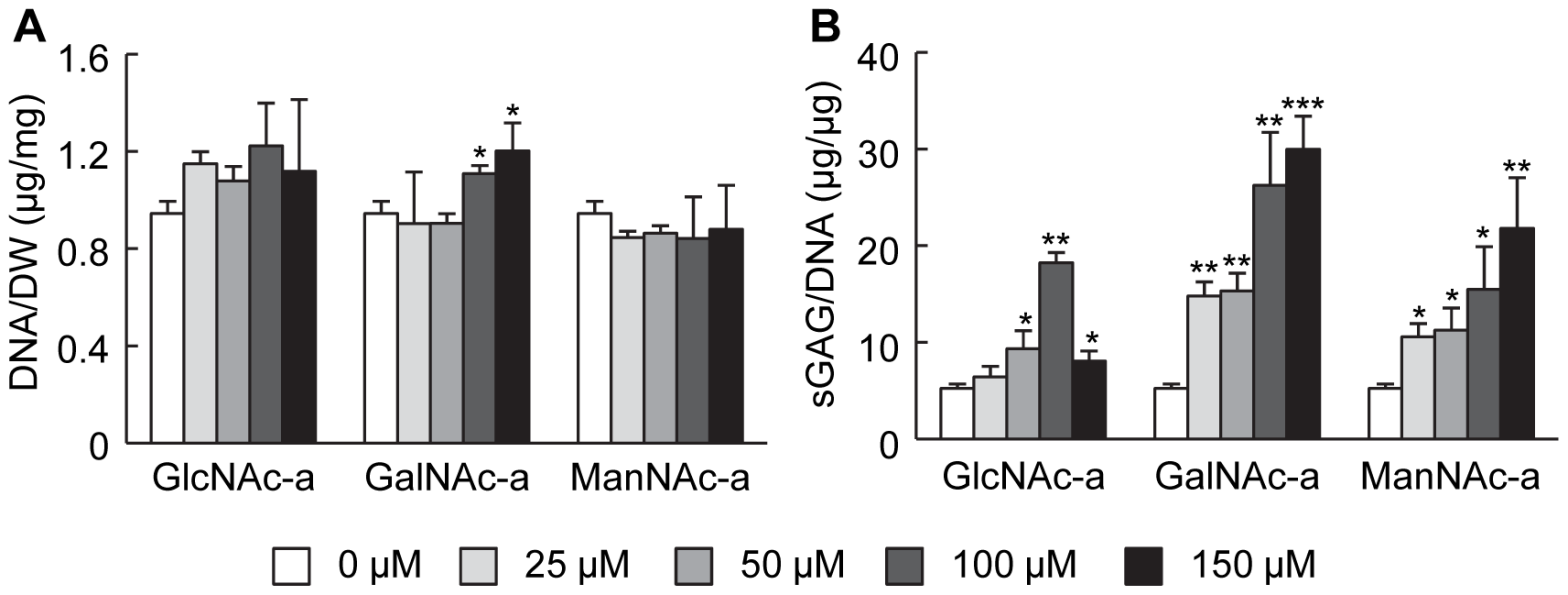
Biochemical analysis of DNA and sGAG for 3D hydrogels of bovine chondrocytes exposed to hexosamine analogs in combination with 10 ng/ml IL-1β. (A) Dry weight of DNA normalized to the construct had minimal variability across all conditions (n = 3). (B) sGAG accumulation, normalized to DNA content, increased with increasing concentrations of hexosamine analog for all conditions except 150 µM GlcNAc-a exposure (n = 3, * P<0.05, **P<0.01, *** P<0.001 versus no analog exposure).

After establishing the concentration range to investigate, we next sought to examine the effects of the hexosamine analogs on ECM accumulation by IL-1β-stimulated chondrocytes. As chondrocytes reside within a three-dimensional (3D) network *in vivo*, a 3D hydrogel culturing system was utilized for these studies (Figure 1D). Also, 3D culture environments minimize chondrocyte dedifferentiation, commonly observed in monolayer cultures [4]. Furthermore, PEGDA hydrogels serve as a “blank slate” with minimal cell and protein interactions to evaluate soluble signals. To model a disease state chondrocytes were exposed to the inflammatory cytokine, IL-1β, for the entire time course of the study. To determine the effect of hexosamine analogs on cell viability after 21 days of exposure within the 3D hydrogels, quantification of the DNA content was performed. DNA quantities were similar across all experimental groups (Figure 2A).

**Figure 3:**
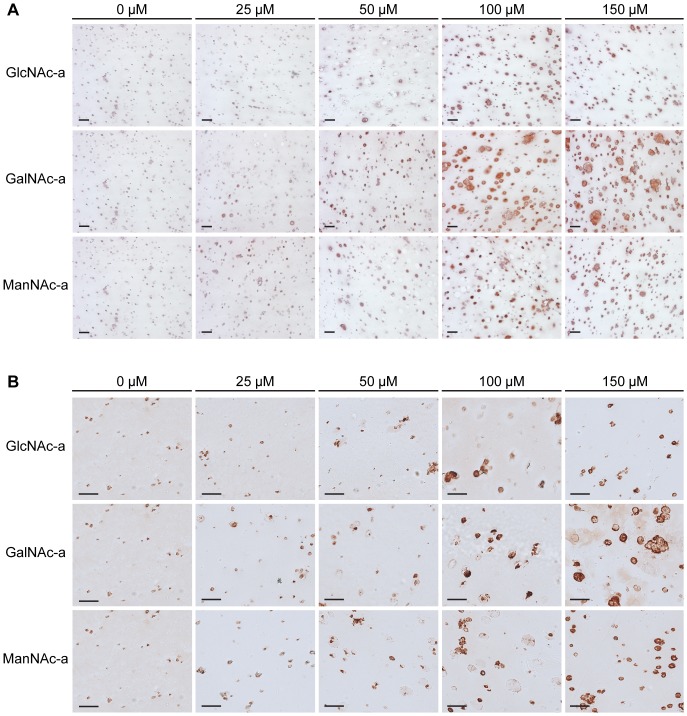
Histological analysis of IL-1β-stimulated chondrocytes exposed to hexosamine analogs in 3D hydrogels. (A) Safranin-O staining for proteoglycans and (B) type II collagen immunostaining (scale bar: 50 µm).

Cytokine stimulation decreases ECM accumulation by chondrocytes in a manner analogous to cartilage degeneration. Therefore, we quantified sGAG accumulation, a predominant ECM molecule in cartilage, within the cell-laden hydrogels using the DMMB dye assay and normalized to the DNA content of these hydrogels to account for variations in cell number between samples. Exposure of the hydrogels containing cytokine-stimulated chondrocytes to the analogs increased sGAG production. Specifically, all of the analogs increased sGAG accumulation in a dose-dependent manner up to 100 µM, with a further increase at 150 µM for GalNAc-a and ManNAc. However in the case of GlcNAc-a exposure, sGAG levels decreased at 150 µM compared to 100 µM (Figure 2B), likely due to reduced metabolic activity accompanying slower proliferation of the cells (Figure S1). The impact of the hexosamine analogs on sGAG production was confirmed via histological staining for proteoglycans using Safranin-O (Figure 3A). An increase in proteoglycan deposition can be observed through 100 µM exposure for all analogs, with a further increase at 150 µM for GalNAc-a and ManNAc-a, as evident by red staining intensity when compared to untreated chondrocytes. To further characterize ECM production, type II collagen deposition in the hydrogels was visualized through use of immunohistochemistry. Analog exposure increased type II collagen deposition, indicated by the red/brown stain, starting at 50 µM for all analogs (Figure 3B) with GalNAc-a exposure resulting in the highest staining intensity. These findings indicate that all three hexosamine analogs have positive effects on stimulating ECM accumulation by IL-1β-stimulated chondrocytes. Furthermore, GalNAc-a exposure induced the greatest cartilage-like tissue formation by the diseased cells, as indicated by increased levels of sGAG content, and proteoglycan and type II collagen staining intensity.

### Altered Gene Expression of IL-1β-Stimulated Chondrocytes Upon Hexosamine Analog Exposure

**Figure 4:**
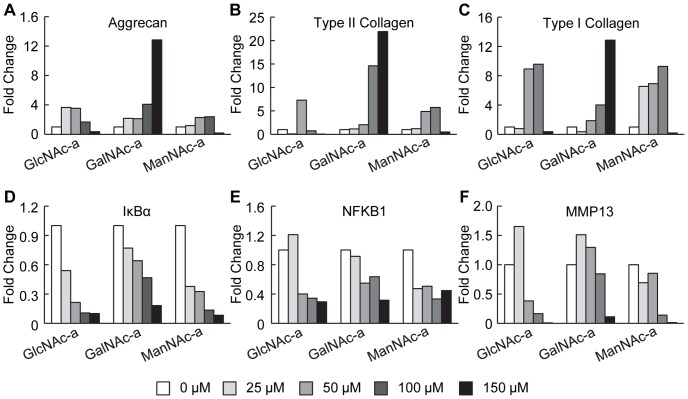
Effects of analog exposure on gene expression of IL-1β-stimulated chondrocytes in 3D hydrogels. Gene expression for ECM proteins (A) aggrecan, (B) type II collagen and (C) type I collagen, and inflammatory proteins (D) IκBα, (E) NFKB1 and (F) MMP13.

Interleukin-1β stimulation of chondrocytes decreases the gene expression levels of ECM proteins [22], [23]. Therefore, we sought to evaluate the gene expression changes of three ECM protein molecules--aggrecan, type II collagen and type I collagen--when chondrocytes were exposed to IL-1β and the hexosamine analogs. Chondrocytes increased gene expression of all three ECM molecules when exposed to each of the hexosamine analogs (Figure 4 A-C), consistent with the increased ECM accumulation observed (Figure 2B and Figure 3).

We further characterized the effects of exposing chondrocytes to the analog library by evaluating gene expression of NFκB transcriptional targets , namely IκBα, NFKB1 and MMP13. All three hexosamine analogs reduced expression of NFKB1 expression (Figure 4) and induced a dose-depedent decreaseIκBα expression (Figure 4E). Gene expression profiles for MMP13 demonstrated an initial maintenance or increase in expression at low concentrations, while at higher concentrations, all analogs decreased MMP13 gene expression (Figure 4F). The MMP13 gene expression response, along with the decrease in IκBα and NFKB1 gene expression provide evidence that all three analogs act via a similar mechanism to alter the cellular behavior in the presence of IL-1β, namely, by reducing NFκB activity.

### Altered ECM Accumulation by Exposure of Chondrogenic Differentiating Mesenchymal Stem Cells to GalNAc-a

Mesenchymal stem cells are an important cell source for cartilage tissue repair after osteochondral trauma, microfracture surgery and in cell-based therapies. Therefore, we sought to evaluate the effects of GalNAc-a on the chondrogenic differentiation of MSCs. As in the chondrocyte experiments, cells were cultured in 3D hydrogels to better mimic the *in vivo* environment and support chondrogenesis (Figure 1E). GalNAc-a was utilized for these studies because it facilitated the greatest recovery from IL-1β-induced matrix loss in chondrocytes. Additionally, dexamethasone, known to inhibit NFκB activity, was used as a control for these experiments. Dexamethasone is also a standard medium supplement for chondrogenic induction of MSCs, as it is thought to be necessary for stem cell differentiation [24], [25]. Finally, we chose to evaluate chondrogenesis in the presence or absence of ascorbic acid, which is required for collagen synthesis and is also an antioxidant that reduces inflammation through radical scavenging [26], [27].

**Figure 5:**
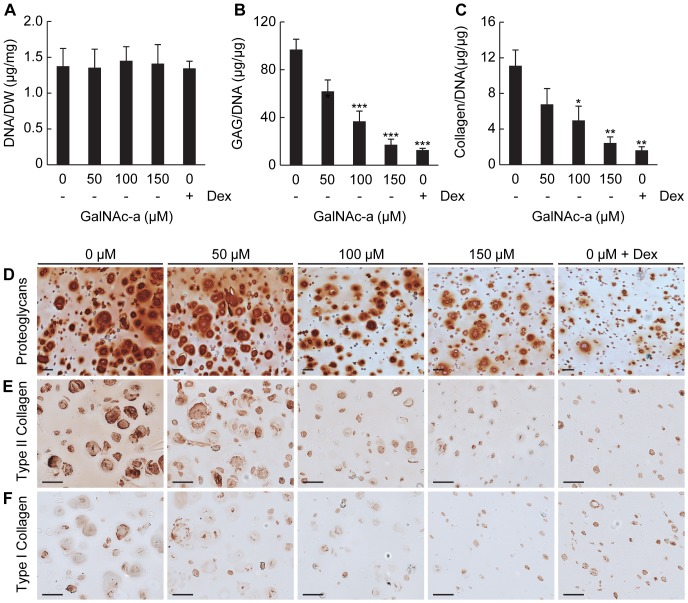
Biochemical analysis of MSCs undergoing chondrogenesis in the presence of ascorbic acid and GalNAc-a or dexamethasone in 3D hydrogels. Quantification of biochemical composition of (A) DNA normalized to construct dry weight, (B) sGAG normalized to DNA content and (C) total collagen normalized to DNA content (n = 4, * P<0.05, ** P<0.01, * P<0.001 versus no analog exposure). (D) Histological staining for proteoglycans using Safranin-O and (E,F) immunohistochemical staining for (E) type II collagen and (F) type I collagen (scale bar: 50 µm).

**Figure 6:**
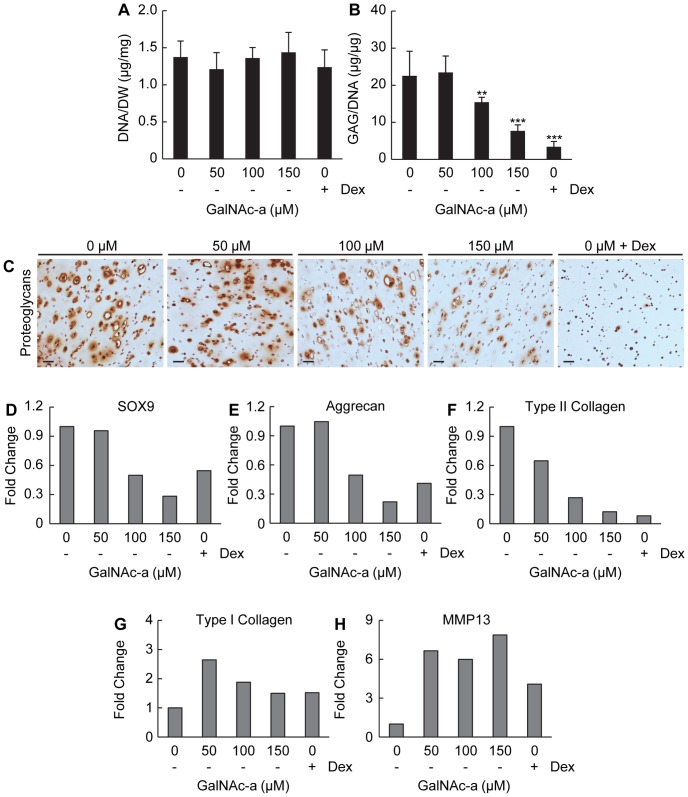
Biochemical and gene expression analysis of MSCs undergoing chondrogenesis (absence of ascorbic acid) in the presence of GalNAc-a or dexamethasone in 3D hydrogels. Quantification of biochemical composition of (A) DNA normalized to construct dry weight and (B) sGAG normalized to DNA content (n = 4, **P<0.01, *** P<0.001 versus no analog exposure). (C) Histological staining for proteoglycans using Safranin-O (scale bar: 50 µm). Gene expression analysis of markers for (D-G) chondrogenesis and matrix production with (H) MMP13 were evaluated (presented as described in Figure Legend 6).

To determine the impact of GalNAc-a exposure on ECM accumulation by chondrogenic-induced MSCs, we evaluated the biochemical content of the cell-laden hydrogels. GalNAc-a exposure did not change the DNA content in any of the groups evaluated (Figure 5A and Figure 6A). ECM accumulation was assessed via sGAG and total collagen quantification. Total collagen accumulation was assayed only in the ascorbic acid-containing medium samples because ascorbic acid is required for collagen synthesis and secretion from cells. GalNAc-a exposure produced a dose-dependent decrease in sGAG in both medium conditions assessed (Figure 5B and Figure 6B). Furthermore, GalNAc-a exposure induced a dose-dependent decrease in total collagen accumulation (Figure 5C). Dexamethasone exposure decreased sGAG accumulation similar to GalNAc-a exposure under both medium conditions (Figure 5B and Figure 6B). Dexamethasone exposure also reduced total collagen accumulation (Figure 5C). The reduction in ECM accumulation was further confirmed via Safranin-O staining for proteoglycans (Figure 5D and Figure 6C), and immunohistochemistry for type II and type I collagen (Figure 5 E,F). These findings indicatethat GalNAc-a inhibits chondrogenic differentiation of the MSCs.

### GalNAc-a Alters Gene Expression of Chondrogenic-Induced Mesenchymal Stem Cells

**Figure 7:**
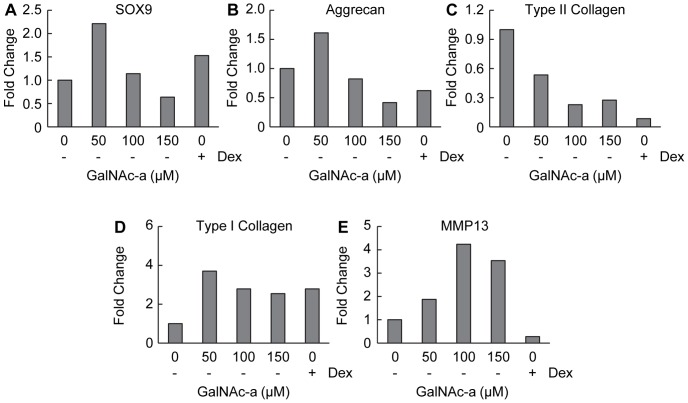
Gene expression analysis of MSCs undergoing chondrogenesis in the presence of ascorbic acid and GalNAc-a or dexamethasone in 3D hydrogels. Markers for (A-D) chondrogenesis and matrix production and (E) MMP13 were evaluated. All data were normalized to individual β-actin levels and presented relative to untreated controls (no analog, no dexamethasone).

We next evaluated gene expression changes related to the chondrogenic markers SOX9, aggrecan and type II collagen, along with type I collagen, a marker for ubiquitous ECM production and fibrocartilage. The chondrogenic transcription factor, SOX9, exhibited a dose-dependent decrease from 50 µM to 150 µM GalNAc-a exposure under both medium conditions (Figure 6D and Figure 7A). Fifty micromolar GalNAc-a exposure increased SOX9 expression in the ascorbic acid-containing medium group (Figure 7A). Aggrecan expression followed a similar trend as SOX9 expression (Figure 6E and Figure 7B). Additionally, GalNAc-a exposure decreased type II collagen expression under both medium conditions similar to the immunohistochemistry results (Figure 6F and Figure 7C). Type I collagen expression initially increased with 50 µM analog exposure and slightly decreased thereafter under both medium conditions, but remained elevated as compared to no analog exposure (Figure 6G and Figure 7D). Dexamethasone exposure decreased the expression of the ECM markers, aggrecan and type II collagen, while increasing expression of type I collagen, similar to the effects of analog exposure (Figure 6 E-G and Figure 7 B-D). Finally, we evaluated gene expression changes for MMP13 after GalNAc-a exposure of chondrogenic-induced MSCs. MMP13 expression increased at all analog concentrations under investigation (Figure 6H and Figure 7E).

### GalNAc-a Alters ECM Accumulation of Chondrogenic-Induced Mesenchymal Stem Cells Stimulated by IL-1β

**Figure 8:**
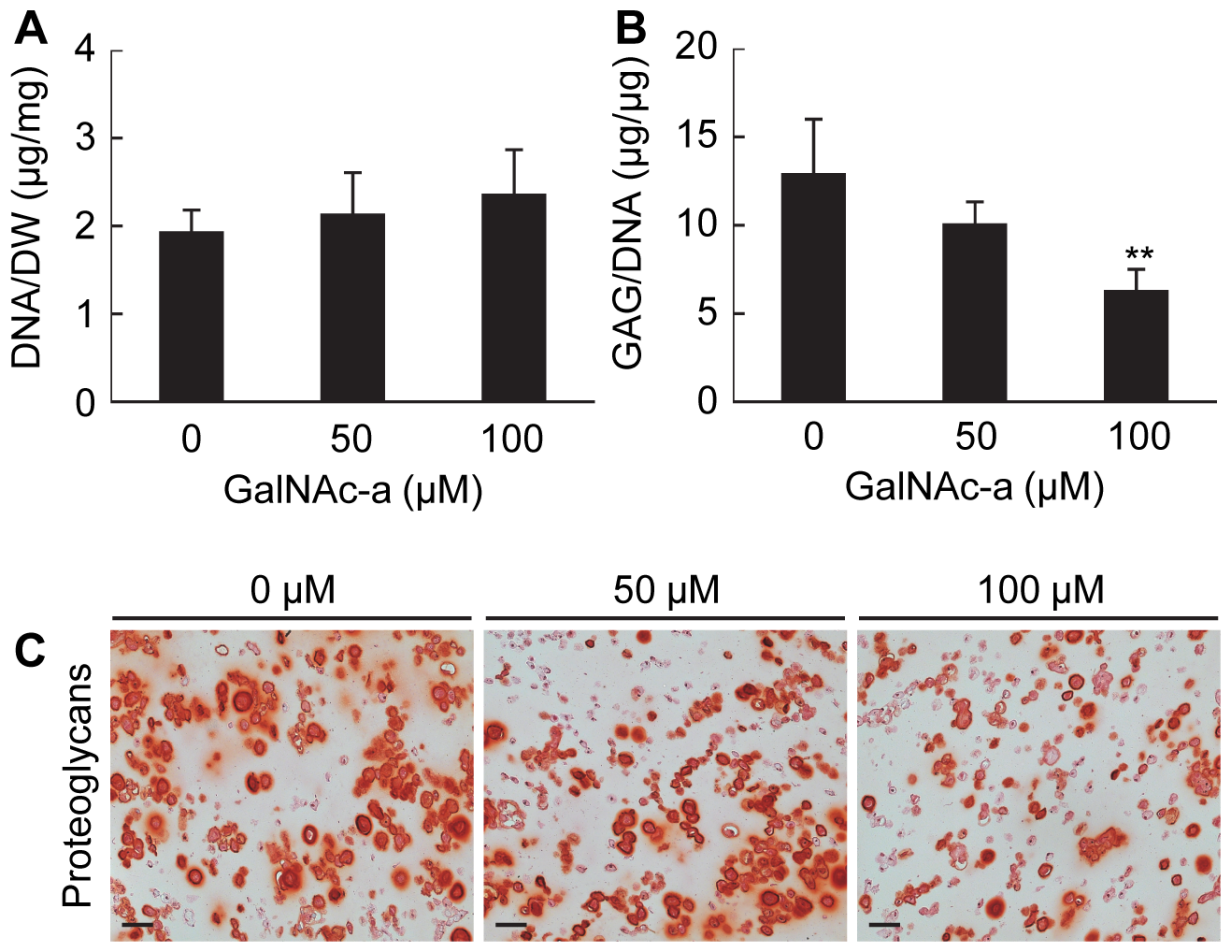
Biochemical analysis of MSCs undergoing chondrogenesis under IL-1β stimulation and GalNAc-a exposure in 3D hydrogels. (A) DNA normalized to construct dry weight and (B) sGAG normalized to DNA content (n = 3, **P<0.01 versus no analog exposure). (C) Histological staining for proteoglycans using Safranin-O (scale bar: 50 µm).

GalNAc-a exposure on chondrogenesis in the presence of IL-1β. IL-1β stimulation of chondrogenic-induced MSCs decreased both DNA content and sGAG accumulation (Figure S2) similar to IL-1β effects alone on chondrocytes [28]. GalNAc-a exposure of IL-1β stimulated chondrogenic-induced MSCs did not have an effect on DNA content (Figure 8A). However, GalNAc-a exposure decreased sGAG accumulation in chondrogenic-induced MSCs exposed to IL-1β (Figure 8 B and C), similar to the trends observed in unstimulated MSCs (Figure 5B).

## Discussion

Current available OA therapeutics target pain management and increased mobility at later stages of tissue damage. There remains a significant need to develop disease-modifying agents that reduce OA progression and restore cartilage homeostasis. Patients who may benefit the most from disease-modifying approaches are those with sports-related or overuse injuries which are strong predictors for OA later in life and those undergoing knee surgery. After injury or surgery there is an increase in inflammatory molecules within the joint space that are purported instigators of OA development. In the first embodiment of this work, we evaluated a potential new drug platform for reversing inflammatory changes in chondrocytes and promoting new tissue formation. The drug platform was composed of the three most common mammalian *N*-acetylated hexosamines (GalNAc, GlcNAc and ManNAc) functionalized on the 3,4,6-position hydroxyl groups with ester-linked butyrate groups. In previous studies, these drug candidates have been shown to inhibit NFκB activity in cancer cells [10], [11]. In the present studies, the newly evaluated analog, GalNAc-a, elicited superior enhancement of ECM accumulation by IL-1β-stimulated chondrocytes. We also expanded the analog evaluation to MSCs, another cell type of interest for cartilage repair. A differential response to the analogs was observed between the IL-1β-stimulated chondrocytes and chondrogenic-induced MSCs.

While the mechanism of action for the analogs is hypothesized to be similar, the therapeutic window differed significantly. We observed that all three analogs increased cartilage-like ECM accumulation, as evident by increased sGAG and type II collagen accumulation. However, only GalNAc-a and ManNAc-a exposure maintained increased ECM accumulation through the highest concentration evaluated; GlcNAc-a exposure exhibited a narrow therapeutic window. Furthermore, GalNAc-a exposure exhibited negligible changes in viability at 150 µM in monolayer cell culture, indicating that GalNAc is the ideal candidate for robust tissue production.

Despite the variation in stimulated tissue production and cytocompatibility, the mechanism of action of all three analogs in IL-1β-stimulated chondrocytes likely share important similarities. All three analogs increased ECM-related gene expression levels, while decreasing IκBα and NFKB1 gene expression. The inhibition of the gene expression of these two proteins by the three analogs is consistent with the reduced NFκB activity reported previously [10], [11]. An increase in type I collagen gene expression is generally considered an indicator of fibrocartilage formation. However, with IL-1β exposure, chondrocytes decrease expression of type I collagen. Therefore, increases in type I collagen are likely not at high enough levels to be an indicator of fibrochondrocytes but that of cells recovering the ability to secrete matrix.

Mesenchymal stem cells are a relevant cell type for cartilage repair, particularly when tissue damage reaches into the subchondral bone. A common surgical technique, microfracture, uses resident MSCs in bone marrow to repair cartilage damage [29], [30]. However, microfracture repair tissue is predominantly composed of mechanically inferior fibrocartilage. There have been reports of hyaline cartilage with the microfracture technique suggesting that the bone marrow-derived MSCs are capable of producing hyaline-like tissue [30], [31]. In our study, GalNAc-a exposure resulted in a dose-dependent decrease in chondrogenic differentiation of MSCs in both the presence and absence of IL-1β stimulation. Dexamethasone exposure evoked a similar response. Dexamethasone is known to inhibit NFκB activity via multiple mechanisms through activation of glucocorticoid receptors (GRs) [32]. One mechanism, transactivation, results in the upregulation of the expression of IκBα, increasing the amount of IκBα present to inhibit NFκB. The other mechanism, transrepression, results in downregulation of NFκB target genes via the activated GR binding to NFκB, rendering it inactive. The specific mechanism of inhibition of chondrogenesis is likely different between dexamethasone and GalNAc-a, although NFκB signaling likely plays a central role in both cases. NFκB signaling plays a crucial role in limb development and chondrogenesis of growth plate chondrocytes. Specifically, growth plate chondrogenesis requires NFκB signaling by inducing BMP-2 expression and activity [33], [34]. BMP signaling is also required for early stages of limb bud development during mesenchymal condensation, an initial embryological stage of chondrogenesis. Kanegae and colleagues showed that NFκB is localized to the most distal region of the developing limb and inhibition of NFκB results in severe alterations in limb growth [35]. Additionally, protein kinase A phosphorylates serine 181 on SOX9, increasing the activity of SOX9 [36]. Protein kinase A is a transcriptional target of NFκB [37]. Ushita and colleagues identified the NFκB protein RelA as a transcriptional inducer of SOX9 expression [38]. Furthermore, transforming growth factor beta (TGF-β) proteins, a family of growth factors that induce chondrogenesis, activate the mitogen-activated protein kinase (MAPK) signaling pathway and NFκB [39], [40]. All of these findings suggest that NFκB plays a central and complex role during chondrogenesis.

Inhibition of SOX9 likely plays a role in the observed reduced chondrogenesis evident by reduced ECM accumulation by MSC exposure to GalNAc-a. SOX9 is the master regulator of chondrogenesis and is known to regulate the expression of type II collagen and aggrecan [41], [42]. During chondrogenesis, expression of SOX9 is regulated by intracellular SMAD proteins which are activated by TGF-β proteins [40], [41]. SOX9 expression decreased starting at 50 µM GalNAc-a exposure, accompanied by decreases in aggrecan and type II collagen gene expression. Additionally, dexamethasone exposure inhibited both aggrecan and type II collagen gene expression, which supports previous literature from two separate research groups that showed inhibition of proteoglycans synthesis in ATDC5 cells, a chondrogenic cell line [43], [44].

The chondrocytes and MSCs were encapsulated and cultured in hydrogels, where minimal direct cell-cell interactions can be established. This lack of direct cell-cell interaction may explain the inhibited chondrogenesis observed in the presence of dexamethasone, which is the opposite of findings from previous studies [24], [25]. Derfoul and colleagues showed that dexamethasone induced chondrogenesis of human MSCs in pellet cultures, where cell-cell contacts are prevalent [24]. Prior studies evaluating cartilage formation in embryos showed dexamethasone was required for inducing cartilage nodule formation in organoid cultured embryos [25]. Furthermore, Zimmermann and colleagues suggested that high density culture, in which intercellular connections of GRs can form, is required for dexamethasone-induced chondrogenesis [25].

Our finding of increased MMP13 expression at low analog concentrations in IL-1β-stimulated chondrocytes and all analog concentrations in chondrogenic-induced MSCs is inconsistent with analog inhibition of NFκB activity. However, regulation of MMP13 expression can be mediated by ECM signaling through type II collagen, and it is possible that these effects were dominant in these studies. In healthy cartilage, the intact pericellular matrix is predominantly composed of proteoglycans, which limits type II collagen binding to discoidin domain receptor 2 (DDR2) [45]. During the early stages of OA, increased ADAMTS (a disintegrin and metalloproteinase gene family) levels result in degradation of proteoglycans, exposing DDRs to collagen. When collagen binds to DDRs, it mediates numerous downstream effects, including cell differentiation, ECM remodeling and cell cycle control [46]. In particular, DDRs have been shown to activate p38 MAPK signaling, which occurs as a result of stress, leading to upregulated MMP13 expression [45], [47], [48]. Altered pericellular matrix composition is a potential mechanism for the increase in MMP13 expression observed in both cell types studied. At low analog concentrations, chondrocytes exhibited an initial increase or maintenance of MMP13 expression, likely due to increased interaction with type II collagen prior to dense pericellular matrix formation. In the chondrogenic induction experiment, the reduction in the proteoglycan-rich pericellular matrix may have increased type II collagen interacting with DDR2, subsequently increasing MMP13 expression.

The NFκB pathway is well conserved across species ranging from the fruit fly to human. Therefore, the differential response to hexosamine analogs between bovine chondrocyte and goat MSCs is unlikely a result of species variance. Within a given cell fate (i.e. chondrocytes or chondrogenesis), the level of response may vary between species as the relative abundance of NFκB protein levels likely varies. Additionally, age-related changes in the degree of response may affect the therapeutic application of these molecules. Therefore, further studies need to be performed to elucidate the degree of therapeutic potential of these molecules in human chondrocytes with varying age and disease.

In summary, we demonstrated the potential of a new class of molecules, C1-OH tributanoylated hexosamines, GalNAc, GlcNAc and ManNAc, to increase cartilage-like tissue accumulation by IL-1β-stimulated chondrocytes. Furthermore, all three molecules reduced NFKB1 and IκBα gene expression, consistent with NFκB inhibitory properties of these analogs. GalNAc-a exposure produced the greatest ECM accumulation by IL-1β-stimulated chondrocytes. However, GalNAc-a exposure produced an opposite effect on MSCs, where a decrease in ECM accumulation was observed. These findings are in support of the function of NFκB signaling during limb development and growth plate chondrogenesis. Our results suggest the potential for a new class of hexosamine analogs as disease-modifying agents for treating cartilage damage, while emphasizing the necessity to investigate multiple means of cartilage repair when developing therapeutic strategies. The *in vivo* effects of the analogs need to be tested to further characterize their therapeutic windows and their effects on multiple cells types in the joint, including, chondrocytes, MSCs, synoviocytes and immune cells.

## Supporting Information

Figure S1 **WST-1 cell proliferation assay for the three analogs investigated.**
(TIF)

Figure S2 **Effect of IL-1β stimulation on biochemical content of chondrogenic-induced MSCs encapsulated in PEGDA hydrogels.** (A) DNA normalized to construct dry weight (n = 3, *P<0.05) and (B) sGAG normalized to DNA content (n = 3, ***P<0.001). (C) Histological staining for proteoglycans using Safranin-O (scale bar: 50 µm).
(TIF)

Table S1 **List of bovine primers used for real-time PCR.**
(DOCX)

Table S2 **List of goat primers used for real-time PCR.**
(DOCX)

## References

[1] Mow VC, Ratcliffe A, Poole AR (1992) Cartilage and diarthrodial joints as paradigms for hierarchical materials and structures. Biomaterials 13: 67–97. 1550898 10.1016/0142-9612(92)90001-5

[2] Pittenger MF, Mackay AM, Beck SC, Jaiswal RK, Douglas R, et al (1999) Multilineage Potential of Adult Human Mesenchymal Stem Cells. Science 284: 143–147. 10102814 10.1126/science.284.5411.143

[3] Kalson NS, Gikas PD, Briggs TWR (2010) Current strategies for knee cartilage repair. International Journal of Clinical Practice 64: 1444–1452. 20716151 10.1111/j.1742-1241.2010.02420.x

[4] Fitzgerald GK, Piva SR, Irrgang JJ (2004) Reports of joint instability in knee osteoarthritis: Its prevalence and relationship to physical function. Arthritis Care Res 51: 941–946. 10.1002/art.20825 15593258

[5] Buckwalter JA (2003) Sports, joint injury, and posttraumatic osteoarthritis. J Orthop Sports Phys Ther 33: 578–588. 14620787 10.2519/jospt.2003.33.10.578

[6] Catterall J, Stabler T, Flannery C, Kraus V (2010) Changes in serum and synovial fluid biomarkers after acute injury (NCT00332254). Arthritis Res Ther 12: R229. 21194441 10.1186/ar3216 PMC3046542

[7] Cameron ML, Fu FH, Paessler HH, Schneider M, Evans CH (1994) Synovial fluid cytokine concentrations as possible prognostic indicators in the ACL-deficient knee. Knee Surg Sports Traumatol Arthrosc 2: 38–44. 7584175 10.1007/BF01552652

[8] Marcu KB, Otero M, Olivotto E, Borzi RM, Goldring MB (2010) NF-kappaB signaling: multiple angles to target OA. Curr Drug Targets 11: 599–613. 20199390 10.2174/138945010791011938 PMC3076145

[9] Aich U, Campbell CT, Elmouelhi N, Weier CA, Sampathkumar SG, et al (2008) Regioisomeric SCFA Attachment to Hexosamines Separates Metabolic Flux from Cytotoxicity and MUC1 Suppression. ACS Chem Bio 3: 230–240. 18338853 10.1021/cb7002708

[10] Campbell CT, Aich U, Weier CA, Wang JJ, Choi SS, et al (2008) Targeting Pro-Invasive Oncogenes with Short Chain Fatty Acid-Hexosamine Analogues Inhibits the Mobility of Metastatic MDA-MB-231 Breast Cancer Cells. J Med Chem 51: 8135–8147. 19053749 10.1021/jm800873k PMC2657678

[11] Elmouelhi N, Aich U, Paruchuri VDP, Meledeo MA, Campbell CT, et al (2009) Hexosamine Template. A Platform for Modulating Gene Expression and for Sugar-Based Drug Discovery. J Med Chem 52: 2515–2530. 19326913 10.1021/jm801661m PMC2721157

[12] Kim TK, Sharma B, Williams CG, Ruffner MA, Malik A, et al (2003) Experimental model for cartilage tissue engineering to regenerate the zonal organization of articular cartilage. Osteoarthritis Cartilage 11: 653–664. 12954236 10.1016/s1063-4584(03)00120-1

[13] Williams CG, Kim TK, Taboas A, Malik A, Manson P, et al (2003) In vitro chondrogenesis of bone marrow-derived mesenchymal stem cells in a photopolymerizing hydrogel. Tissue Eng 9: 679–688. 13678446 10.1089/107632703768247377

[14] Elisseeff J, McIntosh W, Anseth K, Riley S, Ragan P, et al (2000) Photoencapsulation of chondrocytes in poly(ethylene oxide)-based semi-interpenetrating networks. J Biomed Mater Res 51: 164–171. 10825215 10.1002/(sici)1097-4636(200008)51:2<164::aid-jbm4>3.0.co;2-w

[15] Kavalkovich KW, Boynton RE, Murphy JM, Barry F (2002) Chondrogenic differentiation of human mesenchymal stem cells within an alginate layer culture system. In Vitro Cell Dev Biol Anim 38: 457–466. 12605540 10.1290/1071-2690(2002)038<0457:cdohms>2.0.co;2

[16] Lin CC, Anseth KS (2011) Cell-cell communication mimicry with poly(ethylene glycol) hydrogels for enhancing beta-cell function. Proc Natl Acad Sci U S A 108: 6380–6385. 21464290 10.1073/pnas.1014026108 PMC3080983

[17] Yamaoka H, Asato H, Ogasawara T, Nishizawa S, Takahashi T, et al (2006) Cartilage tissue engineering using human auricular chondrocytes embedded in different hydrogel materials. J Biomed Mater Res A 78: 1–11. 16596585 10.1002/jbm.a.30655

[18] Kim Y-J, Sah RLY, Doong J-YH, Grodzinsky AJ (1988) Fluorometric assay of DNA in cartilage explants using Hoechst 33258. Anal Biochem 174: 168–176. 2464289 10.1016/0003-2697(88)90532-5

[19] Farndale RW, Buttle DJ, Barrett AJ (1986) Improved quantitation and discrimination of sulphated glycosaminoglycans by use of dimethylmethylene blue. Biochim Biophys Acta 883: 173–177. 3091074 10.1016/0304-4165(86)90306-5

[20] Woessner JF (1961) The determination of hydroxyproline in tissue and protein samples containing small proportions of this imino acid. Arch Biochem Biophys 93: 440–447. 13786180 10.1016/0003-9861(61)90291-0

[21] Pfaffl MW (2001) A new mathematical model for relative quantification in real-time RT-PCR. Nucleic Acids Res 29: e45. 11328886 10.1093/nar/29.9.e45 PMC55695

[22] Goldring MB, Fukuo K, Birkhead JR, Dudek E, Sandell LJ (1994) Transcriptional suppression by interleukin-1 and interferon-gamma of type II collagen gene expression in human chondrocytes. J Cell Biochem 54: 85–99. 8126089 10.1002/jcb.240540110

[23] Aigner T, McKenna L, Zien A, Fan Z, Gebhard PM, et al (2005) Gene expression profiling of serum- and interleukin-1β-stimulated primary human adult articular chondrocytes – A molecular analysis based on chondrocytes isolated from one donor. Cytokine 31: 227–240. 15955710 10.1016/j.cyto.2005.04.009

[24] Derfoul A, Perkins GL, Hall DJ, Tuan RS (2006) Glucocorticoids promote chondrogenic differentiation of adult human mesenchymal stem cells by enhancing expression of cartilage extracellular matrix genes. Stem Cells 24: 1487–1495. 16469821 10.1634/stemcells.2005-0415

[25] Zimmermann B, Cristea R (1993) Dexamethasone induces chondrogenesis in organoid culture of cell mixtures from mouse embryos. Anat Embryol 187: 67–73. 8430901 10.1007/BF00208197

[26] Sakai A, Hirano T, Okazaki R, Okimoto N, Tanaka K, et al (1999) Large-dose ascorbic acid administration suppresses the development of arthritis in adjuvant-injected rats. Arch Orthop Trauma Surg 119: 121–126. 10392503 10.1007/s004020050374

[27] Conner EM, Grisham MB (1996) Inflammation, free radicals, and antioxidants. Nutrition 12: 274–277. 8862535 10.1016/s0899-9007(96)00000-8

[28] Sun L, Wang X, Kaplan DL (2011) A 3D cartilage – Inflammatory cell culture system for the modeling of human osteoarthritis. Biomaterials 32: 5581–5589. 21565399 10.1016/j.biomaterials.2011.04.028 PMC3109142

[29] Haleem AM, El Singergy AA, Sabry D, Atta HM, Rashed LA, et al (2010) The Clinical Use of Human Culture–Expanded Autologous Bone Marrow Mesenchymal Stem Cells Transplanted on Platelet-Rich Fibrin Glue in the Treatment of Articular Cartilage Defects: A Pilot Study and Preliminary Results. Cartilage 1: 253–261. 21170288 10.1177/1947603510366027 PMC3002255

[30] Kuroda R, Ishida K, Matsumoto T, Akisue T, Fujioka H, et al (2007) Treatment of a full-thickness articular cartilage defect in the femoral condyle of an athlete with autologous bone-marrow stromal cells. Osteoarthritis Cartilage 15: 226–231. 17002893 10.1016/j.joca.2006.08.008

[31] Frisbie DD, Trotter GW, Powers BE, Rodkey WG, Steadman JR, et al (1999) Arthroscopic subchondral bone plate microfracture technique augments healing of large chondral defects in the radial carpal bone and medial femoral condyle of horses. Vet Surg 28: 242–255. 10424704 10.1053/jvet.1999.0242

[32] McMaster A, Ray DW (2007) Modelling the glucocorticoid receptor and producing therapeutic agents with anti-inflammatory effects but reduced side-effects. Exp Physiol 92: 299–309. 17138619 10.1113/expphysiol.2006.036194

[33] Wu S, Flint JK, Rezvani G, De Luca F (2007) Nuclear Factor-κB p65 Facilitates Longitudinal Bone Growth by Inducing Growth Plate Chondrocyte Proliferation and Differentiation and by Preventing Apoptosis. J Biol Chem 282: 33698–33706. 17884819 10.1074/jbc.M702991200

[34] Wu S, Morrison A, Sun H, De Luca F (2011) NF-κB p65 interacts with Stat5b in growth plate chondrocytes and mediates the effects of Growth Hormone on chondrogenesis and on the expression of Insulin-like Growth Factor-1 and Bone Morphogenetic Protein-2. J Biol Chem. 10.1074/jbc.M110.175364 PMC3137048 21592969

[35] Kanegae Y, Tavares AT, Belmonte JCI, Verma IM (1998) Role of Rel/NF-κB transcription factors during the outgrowth of the vertebrate limb. Nature 392: 611–614. 9560158 10.1038/33429

[36] Kumar D, Lassar AB (2009) The Transcriptional Activity of Sox9 in Chondrocytes Is Regulated by RhoA Signaling and Actin Polymerization. Mol Cell Bio 29: 4262–4273. 19470758 10.1128/MCB.01779-08 PMC2715793

[37] Kaltschmidt B, Ndiaye D, Korte M, Pothion S, Arbibe L, et al (2006) NF-kappaB regulates spatial memory formation and synaptic plasticity through protein kinase A/CREB signaling. Mol Cell Bio 26: 2936–2946. 16581769 10.1128/MCB.26.8.2936-2946.2006 PMC1446931

[38] Ushita M, Saito T, Ikeda T, Yano F, Higashikawa A, et al (2009) Transcriptional induction of SOX9 by NF-kappaB family member RelA in chondrogenic cells. Osteoarthritis Cartilage 17: 1065–1075. 19254740 10.1016/j.joca.2009.02.003

[39] Gingery A, Bradley EW, Pederson L, Ruan M, Horwood NJ, et al (2008) TGF-beta coordinately activates TAK1/MEK/AKT/NFκB and SMAD pathways to promote osteoclast survival. Exp Cell Res 314: 2725–2738. 18586026 10.1016/j.yexcr.2008.06.006 PMC2578840

[40] Derynck R, Zhang YE (2003) Smad-dependent and Smad-independent pathways in TGF-[beta] family signalling. Nature 425: 577–584. 14534577 10.1038/nature02006

[41] Furumatsu T, Tsuda M, Taniguchi N, Tajima Y, Asahara H (2005) Smad3 Induces Chondrogenesis through the Activation of SOX9 via CREB-binding Protein/p300 Recruitment. J Biol Chem 280: 8343–8350. 15623506 10.1074/jbc.M413913200

[42] Sekiya I, Tsuji K, Koopman P, Watanabe H, Yamada Y, et al (2000) SOX9 enhances aggrecan gene promoter/enhancer activity and is up-regulated by retinoic acid in a cartilage-derived cell line, TC6. J Biol Chem 275: 10738–10744. 10753864 10.1074/jbc.275.15.10738

[43] Fujita T, Fukuyama R, Enomoto H, Komori T (2004) Dexamethasone inhibits insulin-induced chondrogenesis of ATDC5 cells by preventing PI3K-Akt signaling and DNA binding of Runx2. J Cell Biochem 93: 374–383. 15368363 10.1002/jcb.20192

[44] Owen HC, Miner JN, Ahmed SF, Farquharson C (2007) The growth plate sparing effects of the selective glucocorticoid receptor modulator, AL-438. Mol Cell Endocrinol 264: 164–170. 17182172 10.1016/j.mce.2006.11.006

[45] Vonk LA, Doulabi BZ, Huang C, Helder MN, Everts V, et al (2011) Collagen-induced expression of collagenase-3 by primary chondrocytes is mediated by integrin α1 and discoidin domain receptor 2: a protein kinase C-dependent pathway. Rheumatology 50: 463–472. 21075784 10.1093/rheumatology/keq305

[46] Vogel WF, Abdulhussein R, Ford CE (2006) Sensing extracellular matrix: an update on discoidin domain receptor function. Cell Signal 18: 1108–1116. 16626936 10.1016/j.cellsig.2006.02.012

[47] Xu L, Peng H, Wu D, Hu K, Goldring MB, et al (2005) Activation of the Discoidin Domain Receptor 2 Induces Expression of Matrix Metalloproteinase 13 Associated with Osteoarthritis in Mice. J Biol Chem 280: 548–555. 15509586 10.1074/jbc.M411036200

[48] Xu L, Peng H, Glasson S, Lee PL, Hu K, et al (2007) Increased expression of the collagen receptor discoidin domain receptor 2 in articular cartilage as a key event in the pathogenesis of osteoarthritis. Arthritis Rheum 56: 2663–2673. 17665456 10.1002/art.22761

