# Supplementary figures and images for: Differential Response of Chondrocytes and Chondrogenic-Induced Mesenchymal Stem Cells to C1-OH Tributanoylated N-Acetylhexosamines
Source: PLoS One. 2013 Mar 14;8(3):e58899. doi: 10.1371/journal.pone.0058899 (PMC3597543; doi:10.1371/journal.pone.0058899)

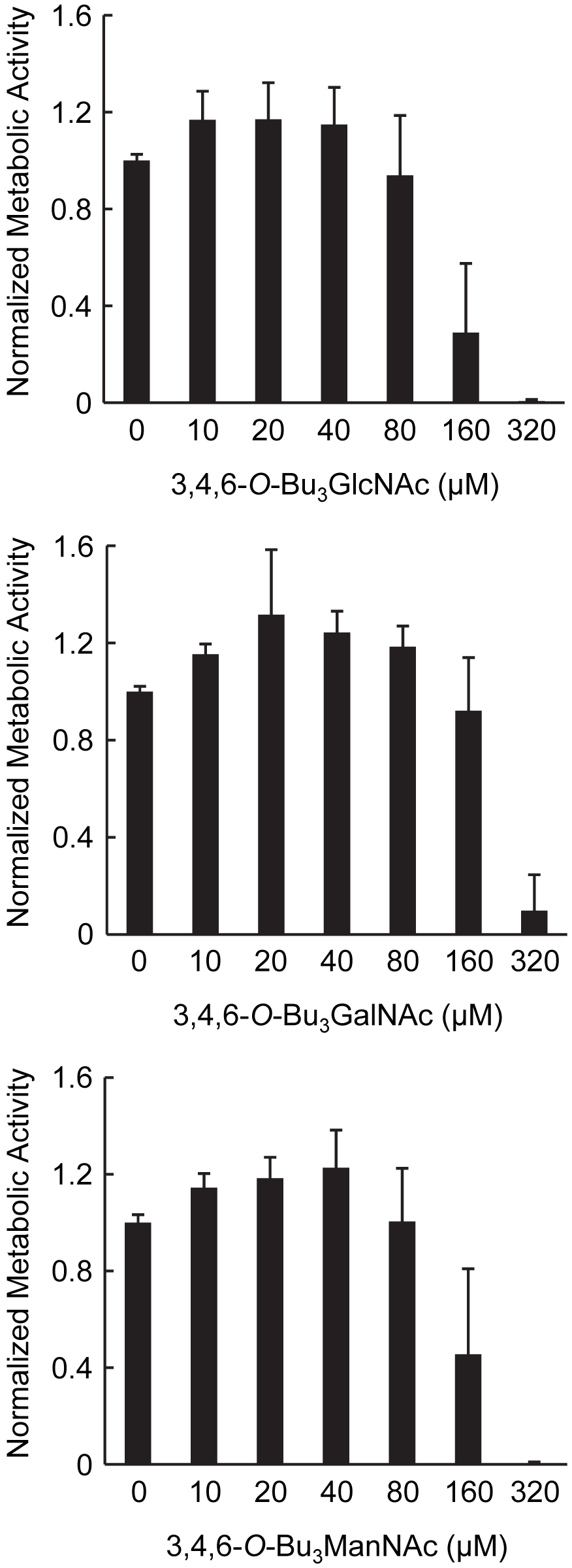

Supplement: Figure S1 — WST-1 cell proliferation assay for the three analogs investigated. (TIF) [file pone.0058899.s001.tif]

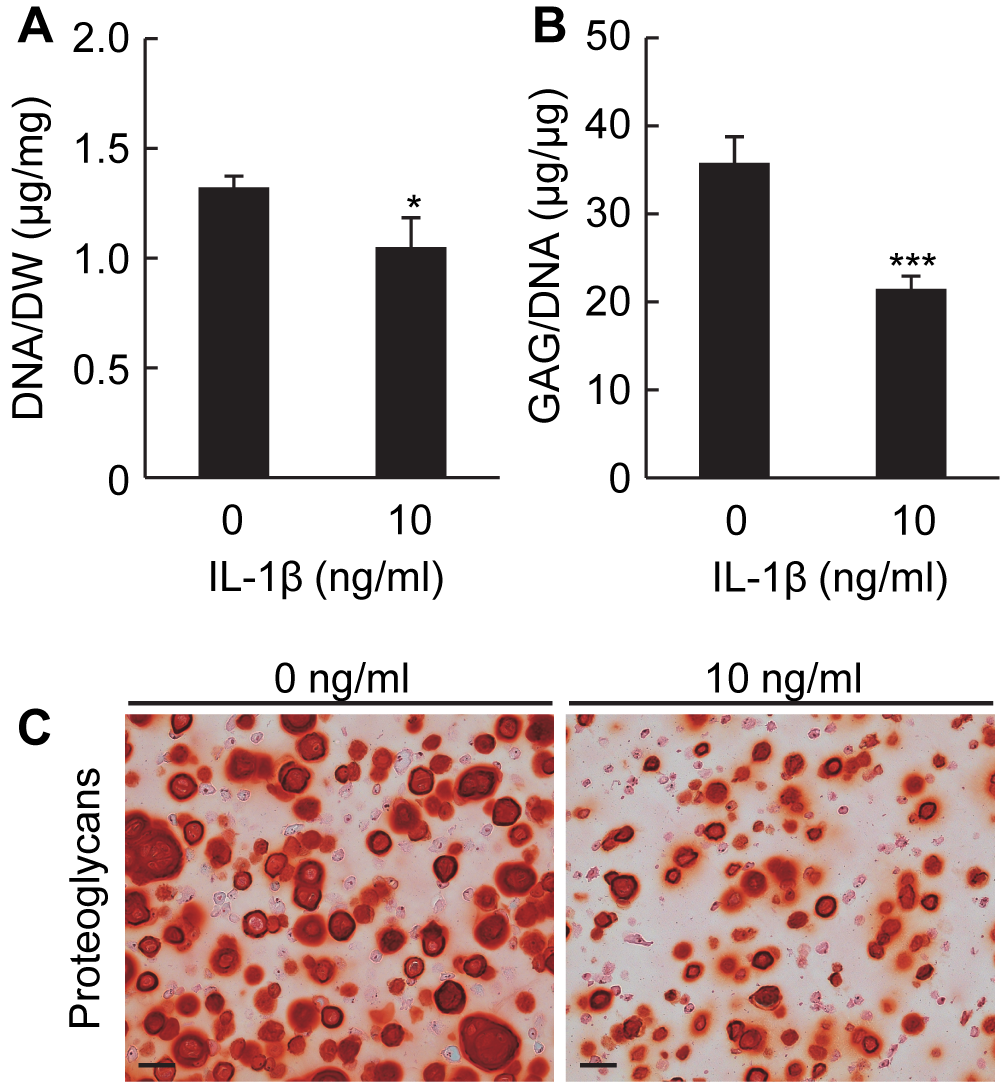

Supplement: Figure S2 — Effect of IL-1β stimulation on biochemical content of chondrogenic-induced MSCs encapsulated in PEGDA hydrogels. (A) DNA normalized to construct dry weight (n = 3, *P<0.05) and (B) sGAG normalized to DNA content (n = 3, ***P<0.001). (C) Histological staining for proteoglycans using Safranin-O (scale bar: 50 µm). (TIF) [file pone.0058899.s002.tif]
