# Supplementary material for: Differential Response of Chondrocytes and Chondrogenic-Induced Mesenchymal Stem Cells to C1-OH Tributanoylated N-Acetylhexosamines
Source: PLoS One. 2013 Mar 14;8(3):e58899. doi: 10.1371/journal.pone.0058899 (PMC3597543; doi:10.1371/journal.pone.0058899)
Supplement: Table S1 — List of bovine primers used for real-time PCR. (DOCX) [file pone.0058899.s003.docx]

**Table S1.** List of bovine primers used for real-time PCR

| **Gene** | **Sequence** |
| --- | --- |
| **Aggrecan** | F-F-CATCGGGCTTGCCAGAGTT  R-ACTGGTGTCCACGAACGTAATG |
| **Type I Collagen** | F-GGGCAACAGCAGATTCACTTAC  R-CAAGGATAGGCAGGCGAGAT |
| **Type II Collagen** | F-GCAACCCTGGAACTGATGGA  R-GCTCACCCGTTTGACCTTTT |
| **IκBα** | F-GCAGGCCACCAACTACAATG  R-AGTGACACCAGGTCGGGATT |
| **NFκB1** | F-TTACAAAACCAGCCTCCGTG  R-GCCGAAACTGTCCGAGAAA |
| **MMP13** | F-GCTCACGCTTTCCCTCCT  R-CAAACTCATGGGCAGCAACA |
| **Beta Actin** | F-TGGCACCACACCTTCTACAATGAGC  R-GCACAGCTTCTCCTTAATGTCACGC |
