# Supplementary material for: Differential Response of Chondrocytes and Chondrogenic-Induced Mesenchymal Stem Cells to C1-OH Tributanoylated N-Acetylhexosamines
Source: PLoS One. 2013 Mar 14;8(3):e58899. doi: 10.1371/journal.pone.0058899 (PMC3597543; doi:10.1371/journal.pone.0058899)
Supplement: Table S2 — List of goat primers used for real-time PCR. (DOCX) [file pone.0058899.s004.docx]

**Table S2.** List of goat primers used for real-time PCR

| **Gene** | **Sequence** | **Efficiency (%)** |
| --- | --- | --- |
| **Aggrecan** | F-CACGATGCCTTTCACCACGAC R-TGCGGGTCAACAGTGCCTATC | 90 |
| **Type I Collagen** | F-AGGGCCAAGACGAAGACATC R-AGATCACGTCATCGCACAACA | 84 |
| **Type II Collagen** | F-GTGGAGCAGCAAGAGCAAGGA R-CTTGCCCCACTTACCAGTGTG | 63 |
| **SOX9** | F-TTCATGAAGATGACCGACGA R-CACACCATGAAGGCGTTCAT | 90 |
| **MMP13** | F-GCTCACGCTTTCCCTCCT  R-CAAACTCATGGGCAGCAACA | 97 |
| **Beta Actin** | F-TGGCACCACACCTTCTACAATGAGC R-GCACAGCTTCTCCTTAATGTCACGC | 80 |
